# Employing plasma proteins in proteomic Mendelian randomization analysis to identify therapeutic targets for duodenal ulcer

**DOI:** 10.1097/MD.0000000000045093

**Published:** 2025-10-31

**Authors:** Xu Luo, Dan Luo, Chenhao Liu, Huize Zhang, Mingyue Long, Simin Cao, Yi Liu

**Affiliations:** a Clinical College, Chengdu University of Traditional Chinese Medicine, Chengdu, Sichuan, China; b School of Basic Medicine, Chengdu University of Traditional Chinese Medicine, Chengdu, Sichuan, China.

**Keywords:** drug targets, duodenal ulcer, Mendelian randomization, plasma protein

## Abstract

Proteomics serves as a primary source of therapeutic targets. In this study, we performed a Mendelian randomization (MR) analysis within the proteomic scope to identify candidate protein markers and potential therapeutic targets for duodenal ulcer (DU). This study utilized MR and co-localization analysis within the proteomic framework. Data on 2088 plasma proteins were carefully collected from a study that detected 4907 protein quantitative trait loci. The genetic association data for DU were sourced from the UK Biobank, which encompassed 1908 cases and 461,025 controls. MR used single nucleotide polymorphisms as a genetic tool to estimate the causal effects of exposure on outcomes, in order to screen candidate proteins associated with DU. Meanwhile, Bayesian co-localization analysis is used to determine the probability of shared causal genetic variation between features. Additionally, 2-step MR was employed to quantify the proportion of protein-mediated risk factors for DU. Finally, protein-protein interaction analysis was conducted to elucidate the potential link between proteins and drugs currently used for treating DU. Using the Drug Signature Database, potential targeted drugs for druggable proteins were explored. We identified 11 plasma proteins that were significantly associated with DU. Elevated levels of FLT4, IGSF3, IL6ST, EPHB4, DPEP2, SEMA6A, and IL1R1 were found to have a risk-conferring effect. Conversely, increased levels of REG1B, GOLM1, FAM3D, and QSOX2 exhibited a protective effect. Notably, none of these 11 proteins demonstrated evidence of reverse causality. Bayesian co-localization analysis indicated that REG1B, FLT4, GOLM1, EPHB4, and FAM3D shared the same genetic variations as those associated with DUs. Additionally, the protein target IL1R1, which is related to DU drugs, and 6 pharmaceutically relevant proteins, namely REG1B, IL6ST, FLT4, DPEP2, QSOX2, and EPHB4, were identified. Our research found that REG1B, FLT4, IGSF3, IL6ST, GOLM1, EPHB4, DPEP2, FAM3D, QSOX2, SEMA6A, and IL1R are associated with DU. Among them, IL1R1, REG1B, IL6ST, FLT4, DPEP2, QSOX2, and EPHB4 may become drug targets for further clinical research on DU. Targeting these proteins during drug development may provide a preferred and cost-effective approach for treating DU.

## 1. Introduction

Peptic ulcer, a lesion typically found in the stomach and duodenum,^[1]^ ranks among the most prevalent gastrointestinal diseases. In the general population, the incidence rate among middle-aged individuals ranges from 0.1% to 0.3%, and the lifetime prevalence is between 5% and 10%.^[2]^ Duodenal ulcer (DU), a component of the broader peptic ulcer disease is defined as the disruption of the integrity of the proximal small intestinal mucosa, resulting in open lesions.^[3]^ The long-term or high-dose use of nonsteroidal anti-inflammatory drugs, abnormal gastric acid secretion, *Helicobacter pylori* infection, genetic factors, and lifestyle habits can all potentially impair mucosal function. This impairment leads to gastrointestinal mucosal inflammation, tissue necrosis, and ultimately, ulcer formation.^[4,5]^ Gastrointestinal diseases, with DU being no exception, are highly prevalent in Western countries. They consume substantial healthcare resources, impose a heavy socioeconomic burden, and significantly impact the quality of life of the affected individuals.^[6]^ Although clinical studies have been carried out from various perspectives to gain a better understanding of DU for more effective prevention and management, the proteomic etiology of DU remains to be comprehensively explored. Proteomic research not only deepens our molecular-level understanding but also aids in uncovering potential therapeutic targets. This is because many circulating plasma proteins often serve as key regulators in molecular pathways.^[7]^

Genome-wide association study (GWAS) aim to unearth genetic variations associated with phenotypes through genomic comparisons.^[8]^ Recent genetic association analyses of the plasma proteome have enabled the systematic evaluation of the causal consequences of changes in plasma protein levels.^[9]^ Identifying potential proteins associated with DU is crucial as it helps us decipher the underlying mechanisms of the disease and pinpoint relevant drug targets. The Mendelian randomization (MR) design, an epidemiological approach, strengthens causal inference by using genetic variations as instrumental variables (IVs) for exposure, such as protein-cycling levels. Given that exposure IVs are randomly allocated during pregnancy and are unlikely to be influenced by disease status, MR studies can rule out unobserved confounding factors and reverse causality, thus overcoming typical pitfalls in observational studies.^[10]^

In this study, we conducted a protein-wide MR study based on a large volume of blood protein data from a large-scale study, complemented by co-localization analysis, to explore plasma proteins associated with DU. Subsequently, we evaluated the causal relationship between plasma proteins and risk factors for DU and quantified the proportion of protein-mediated effects of these risk factors on DU. Finally, we constructed a protein-protein interaction (PPI) network to link the identified proteins with the current drug targets for DU and assess the druggability of the identified proteins. Unraveling the complex mechanisms by which these proteins influence the onset of DU and evaluating their druggability provides valuable insights for the development of innovative treatment strategies for DU.

## 2. Research design and methods

### 2.1. Research design

**Figure 1.**
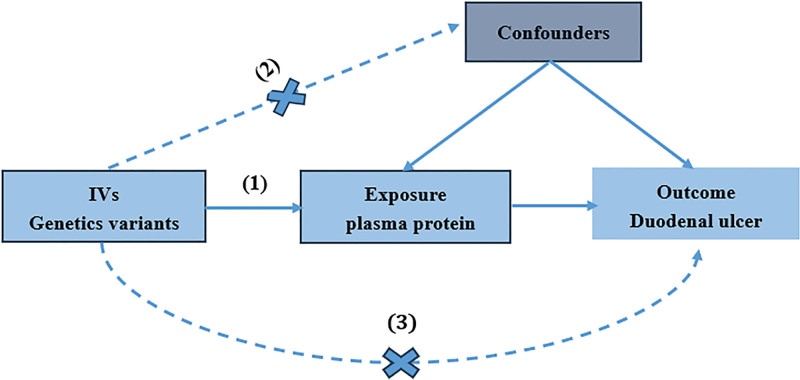
Schematic representation of the Mendelian randomization analysis. The continuous lines represent the relationships that hold in Mendelian randomization analysis. Broken lines represent associations that violate the assumptions of Mendelian randomization. IVs = instrumental variables.

The objective of this study is to identify potential therapeutic targets for DU by means of plasma proteins and to assess the druggability of the identified proteins, thereby laying a solid foundation for the precise prevention and treatment of DU in the future. Firstly, MR analysis was carried out to detect plasma proteins associated with DU. To obtain reliable results, MR must meet 3 key assumptions^[11]^: the genetic variants employed in the analysis should exhibit a significant association with the exposure; the genetic variants extracted as IVs for exposure should be independent of the confounding factors related to the selected exposure and outcome; the genetic variants should influence the outcome solely through the exposure and not through other biological pathways. As depicted in Figure 1.

**Figure 2.**
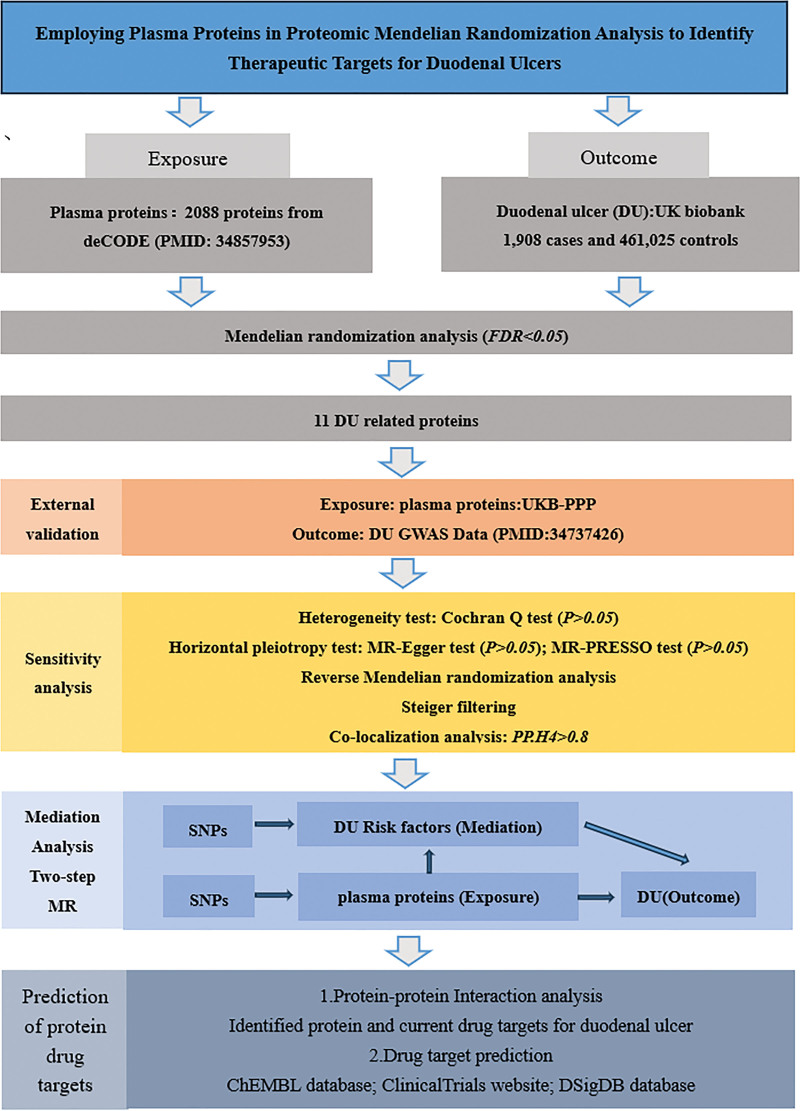
The specific flowchart of Mendelian randomization study design. DU = duodenal ulcer, FDR = false discovery rate, GWAS = genome-wide association study, MR = Mendelian randomization, SNPs = single nucleotide polymorphisms.

The plasma protein data was sourced from the published deCODE database, while the DU data was obtained from the UK Biobank. Subsequently, external validation, the Steiger direction test, Bayesian co-localization, and bidirectional MR were utilized for sensitivity analysis to demonstrate the robustness of the results. Employing a 2-step MR method, mediation analysis was conducted to quantify the impact of co-located proteins on DU via risk factors. Finally, PPI and the identified protein targets were predicted to determine the pharmacological targets of DU and the druggability of the identified proteins. The process is illustrated in Figure 2. Since all data are derived from publicly available sources and informed consent and approval have already been obtained, this study does not necessitate further informed consent or ethical approval.

### 2.2. Data source

#### 2.2.1. Proteomic data source

The proteomic data was sourced from a study carried out by Ferkingstad et al.^[12]^ This study conducted GWASs on plasma protein levels in 35,559 Icelandic individuals, and 4907 protein quantitative trait loci (pQTL) were detected. We collected 2088 plasma proteins for research. These plasma proteins exclude strong linkage disequilibrium (LD) (*r*^2^ = 0.001 and kb = 10,000). Set the filtering threshold to *P* < 5 × 10^−8^ and include it in MR analysis.^[13]^ (Please refer Table S1, Supplemental Digital Content, https://links.lww.com/MD/Q306 for details.)

#### 2.2.2. Source of results data

The result data used for DU in this study came from the GWAS study of the UK Biobank, in which 462,933 Europeans (1908 cases and 461,025 controls) were analyzed and 9,851,867 single nucleotide polymorphisms (SNPs) were identified.

#### 2.2.3. External validation Data

In addition, we used plasma pQTL data from Sun et al’s study^[14]^ which included 2923 plasma proteins from 33,469 participants, as well as GWAS data for DU obtained from Jiang et al’s^[15]^ genome-wide genotyping array study (n_case_ = 1484, n_control_ = 454,864) for external validation (see Table 1 for details).

**Table 1:** Data sources used in this study.

Datasets	Sample size	Population	Sources
Exposure (plasma protein)			
deCODE (primary)	4907	Icelander	Ferkingstad et al^[12]^ (PMID: 34857953)
UKB-PPP (validation)	33,469	European	Sun et al^[14]^ (PMID: 37794186)
Outcomes			
Duodenal ulcer (primary)	462,933	European	UK Biobank
Duodenal ulcer (validation)	456,348	European	Jiang et al^[15]^ (PMID: 34737426)
Mediation			
Treatment/medication code: ibuprofen	337,159	European	UK Biobank
Alcoholic drinks per week	335,394	European	Liu et al^[16]^ (PMID: 30643251)
Poor diet	334,075	European	Carey et al^[17]^ (PMID: 38965376)
Seen a psychiatrist for nerves, anxiety, tension, or depression	460,702	European	UK Biobank
Types of physical activity in last 4 weeks: other exercises (e.g., swimming, cycling, keep fit, bowling)	460,376	European	UK Biobank
Body mass index (BMI)	454,884	European	UK Biobank
Current tobacco smoking	462,434	European	UK Biobank

#### 2.2.4. Mediation data

The data of treatment/medication code: ibuprofen in this study came from the GWAS study of the UK Biobank, in which 337,159 Europeans (41,952 cases and 295,207 controls) were analyzed and 10,894,596 SNPs were identified. The data of alcoholic drinks per week came from Liu et al’s study,^[16]^ in which 335,394 Europeans were analyzed and 11,887,865 SNPs were identified, The data of poor diet came from Carey et al’s study,^[17]^ in which 337,045 Europeans were analyzed and 13,176,603 SNPs were identified. The data of seen a psychiatrist for nerves, anxiety, tension, or depression in this study came from the GWAS study of the UK Biobank, in which 460,702 Europeans (53,414 cases and 407,288 controls) were analyzed and 9,851,867 SNPs were identified. The data of types of physical activity in last 4 weeks: other exercises (e.g., swimming, cycling, keep fit, bowling) in this study came from the GWAS study of the UK Biobank, in which 460,376 Europeans (222,470 cases and 237,906 controls) were analyzed and 9,851,867 SNPs were identified. The data of body mass index (BMI) in this study came from the GWAS study of the UK Biobank, in which 454,884 Europeans were analyzed and 9,851,867 SNPs were identified. The data of current tobacco smoking in this study came from the GWAS study of the UK Biobank, in which 462,434 Europeans were analyzed and 9,851,867 SNPs were identified. See Table 1 for details.

### 2.3. Statistical analysis

#### 2.3.1. MR analysis

Perform 2 sample MR analysis using R software (version 4.3.1; originating from the University of Auckland, New Zealand) and TwoSampleMR package (version 0.6.8), gwasglue package (version 0.0.90000), gwasvcv package (version 0.1.1), and MR-PRESSO package (version 1.0.).

In the primary MR analysis, plasma proteins were designated as the exposure, and DU was set as the outcome. When only a single pQTL was available as an IV for plasma proteins, the Wald ratio method was employed to assess the causal effect.^[18]^ If 2 or more IVs are available, we use inverse variance weighted (IVW), MR Egger, weighted median, simple mode, and weighted mode to estimate causality. This study applies the classic IVW to the primary MR analysis, which provides a consistent estimation of the causal effects of exposure on the results.^[19]^ The statistical results are presented in the form of odds ratio (OR) and 95% confidence interval (95% CI). We used false discovery rate (FDR) correction to control for *P*, and setting *P*_FDR_ < .05 after FDR correction was considered statistically significant.^[20]^

#### 2.3.2. Sensitivity analysis

We calculated the *Q* statistic to assess the presence of heterogeneity among the tools. If the *Q* value is significant (*P* < .05), we employed a multiplicative random-effects model based on IVW. Conversely, when *P* > .05, indicating no heterogeneity, a fixed-effects model was utilized.^[21]^ And further apply MR Egger intercept data to evaluate horizontal pleiotropy, with *P* > .05 indicating no horizontal pleiotropy.^[22]^ MR-PRESSO method is used to test for horizontal pleiotropy and correct for it by excluding outlier variables; the *P* > .05 of the global test indicates the absence of horizontal pleiotropy.^[23]^

To strengthen the robustness of the causal relationship, we performed bidirectional MR analysis, with DU designated as the exposure and pQTL as the outcome. This approach enables the detection of potential reverse causal relationships, mitigates the impact of confounding factors, and ensures the integrity of our genetic association studies.^[24]^ Furthermore, we carried out Steiger orientation tests to ascertain the direction of the association between the identified proteins and diseases. The Steiger filtering is based on the assumption that valid IVs should account for more changes in the exposure than in the outcome. If an IV meets this criterion, its direction is labeled as “TRUE”; otherwise, it is designated as “FALSE.” After excluding SNPs with “FALSE” directions, we replicated all MR analyses using the IVW method and *P*_FDR_ < .05 was considered statistically significant.^[25]^

#### 2.3.3. Co-localization analysis

Co-localization is a further analysis that strengthens the results of genetic research by searching for evidence of the same genetic variations associated with exposure and outcomes. This helps to confirm that genetic variations are indeed causally related to outcomes, rather than being the result of LD or other confounding factors. We used the coloc R package^[26]^ for co-localization analysis to determine whether the identified association between known proteins and DU is driven by LD. Bayesian analysis evaluated the support for 5 mutually exclusive hypotheses: PPH0: Is genetic variation unrelated to any trait; PPH1: Related to only one feature; PPH2: Only related to another feature; PPH3: Related to these 2 characteristics, but with different causal variations; PPH4: Associated with these 2 traits and sharing the same causal variation.^[27]^ We computed the posterior probability for each hypothesis and determined the presence of evidence for protein co-localization based on the protein-based posterior probability. If the posterior probability of the common causal variation hypothesis PPH4 ≥ 0.8, it is considered that the 2 signals have strong co-localization support.^[28]^

#### 2.3.4. Mediation analysis

Through an extensive literature review, the risk factors associated with the onset of DU have been identified. These include *H pylori* infection, long-term use of nonsteroidal anti-inflammatory drugs, excessive gastric acid secretion, genetic susceptibility, long-term smoking and alcohol consumption, mental stress and anxiety, an unhealthy diet, physical exercise levels, BMI, etc.^[29–32]^ We conducted a mediation analysis by incorporating these risk factors and using a 2-step MR method to quantify the impact of identified proteins on DU. The overall effect of exposure on the results can be divided into direct and indirect effects. In our study, the total effect of identified proteins on DU includes: the direct effect of identified proteins on DU, calculated by first-order MR; estimate the indirect effects mediated by mediators using the product method.^[33]^ The detailed information on GWAS summary data on risk factors for DU is shown in Table 1.

#### 2.3.5. Drug protein identification and PPI network

To evaluate the pharmacological properties of the identified proteins, we used the Open Targets Platform (https://platform.opentargets.org/) and use the search term “DU” to obtain drugs and drug targets related to DU.^[34]^ Next, we will use the string database version 12.0 (https://cn.string-db.org/) to explore PPI networks, and use Cytoscape software to visualize the interactions between identified targets and approved DU drug targets.^[35]^ In addition, we evaluated the drug potential of candidate target proteins by referring to Finan’s study of 4479 potential drug genes, which can be classified into 3 levels: Tier 1. This tier incorporated the targets of approved drugs and drugs in clinical development. Tier 2. This tier incorporated proteins closely related to drug targets or with associated drug-like compounds. Tier 3. This tier incorporated extracellular proteins and members of key drug target families.^[36]^ Search for clinical trials (https://www.ClinicalTrials.gov); collect clinical stage data of target proteins under development; obtain information on the compound name, molecular type, and action type of the target protein clinical drug through the ChEMBL database (https://www.ebi.ac.uk/chembl).^[37]^ Finally, using the Enrichr platform (https://maayanlab.cloud/Enrichr/) as an accessible resource for collecting and integrating massive amounts of gene function information, it is possible to discover potential targeted drugs for druggable proteins based on the Drug Signature Database (DSigDB).^[38,39]^

## 3. Result

### 3.1. MR analysis

**Figure 3.**
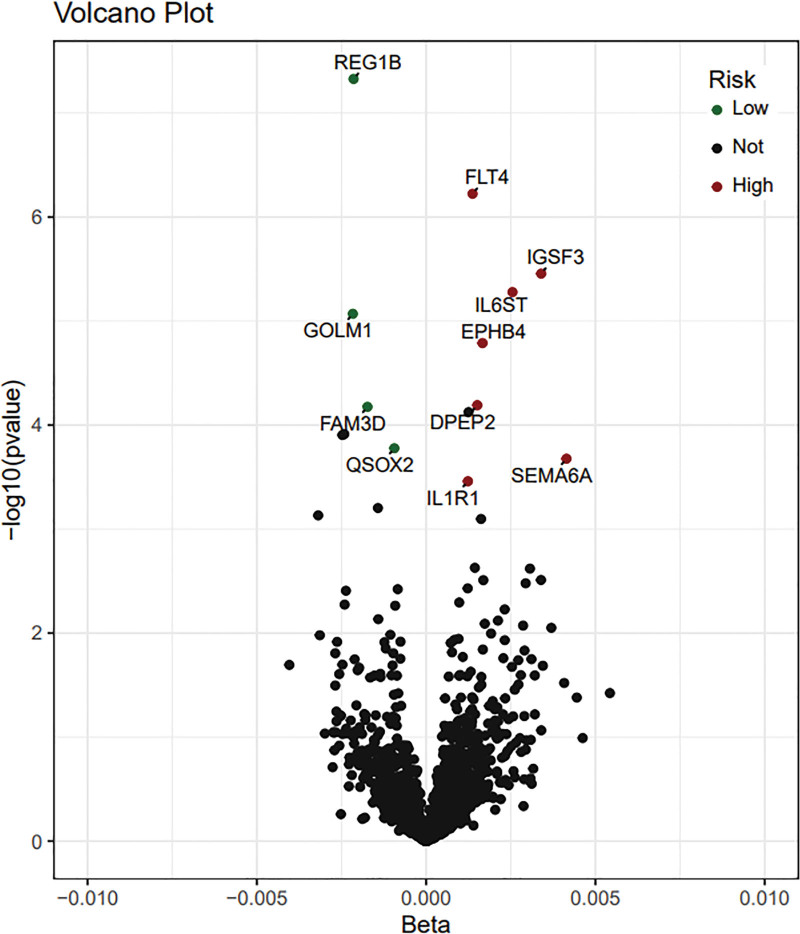
Volcanic diagram of plasma protein and MR results of DU. The plasma proteins represented by the red dots are positively correlated with DU, the plasma proteins represented by the green dots are negatively correlated with DU, and the plasma proteins represented by the black dots are not correlated with DU. DU = duodenal ulcer, MR = Mendelian randomization.

**Figure 4.**
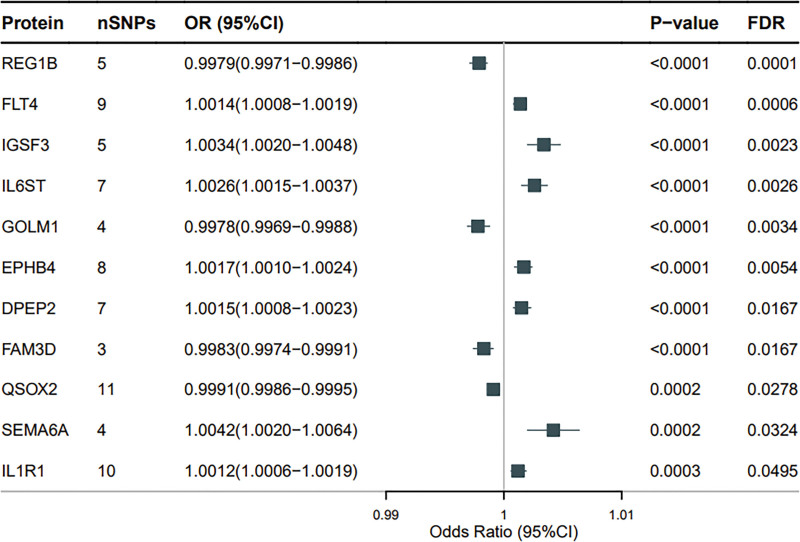
Forest plot of 11 plasma proteins associated with DU analyzed by MR. A square is a causal estimate of OR values, and the horizontal line on the square represents the 95% confidence interval of these OR values. nSNPs: used to estimate the number of SNPs for causal effects in the graph. The *P*-value was determined by IVW nuclear magnetic resonance method. CI = confidence interval, DU = duodenal ulcer, IVW = variance weighted reciprocal, MR = Mendelian randomization, OR = odds ratio, SNPs = single nucleotide polymorphisms.

MR analysis identified 11 plasma proteins associated with DU, including REG1B, FLT4, IGSF3, IL6ST, GOLM1, EPHB4, DPEP2, FAM3D, QSOX2, SEMA6A, and IL1R1 (Figs. 3 and 4 and Figure S1, Supplemental Digital Content, https://links.lww.com/MD/Q305). Specifically, FTL4 (OR = 1.0014, 95% CI = 1.0008–1.0019, *P* = 5.98E−07, *P*_FDR_ = 5.98E−05), IGSF3 (OR = 1.0034, 95% CI = 1.0020–1.0048, *P* = 3.50E−06, *P*_FDR_ = 0.0023), IL6ST (OR = 1.0026, 95% CI = 1.0015–1.0037, *P* = 5.27E−06, *P*_FDR_ = 0.0026), EPHB4 (OR = 1.0017, 95% CI = 1.0010–1.0024, *P* = 1.63E−05, *P*_FDR_ = 0.0054), DPEP2 (OR = 1.0015, 95% CI = 1.0008–1.0023, *P* = 6.44E−05, *P*_FDR_ = 0.0167), QSOX2 (OR = 0.9991, 95% CI = 0.9986–0.9995, *P* = .0002, *P*_FDR_ = 0.0278), SEMA6A (OR = 1.0042, 95% CI = 1.0020–1.0064, *P* = .0002, *P*_FDR_ = 0.0324), and IL1R1 (OR = 1.0012, 95% CI = 1.0006–1.0019, *P* = .0003, *P*_FDR_ = 0.0495) are positively correlated with DU. REG1B (OR = 0.9979, 95% CI = 0.9971–0.9986, *P* = 4.71E−08, *P*_FDR_ = 9.41E−05), FAM3D (OR = 0.9983, 95% CI = 0.9974–0.9991, *P* = 6.68E−05, *P*_FDR_ = 0.0617), GOLM1 (OR = 0.9978, 95% CI = 0.9969–0.9988, *P* = 8.52E−06, *P*_FDR_ = 0.0034), and QSOX2 (OR = 0.9991, 95% CI = 0.9986–0.9995, *P* = .0002, *P*_FDR_ = 0.0278) are negatively correlated with DU.

Our main findings have been validated in other datasets, with REG1B (OR = 0.7184, 95% CI = 0.5723–0.9017, *P* = .0043) reducing the risk of DU; FLT4 (OR = 1.1751, 95% CI = 1.0239–1.3487, *P* = .0217), EPHB4 (OR = 1.3293, 95% CI = 1.1105–1.5911, *P* = .0019), and DPEP2 (OR = 1.4325, 95% CI = 1.0808–1.8986, *P* = .0124) are positively correlated with DU, while other proteins are not related to DU. QSOX2, GOLM1, and SEMA6A proteins cannot be found in the UKBPPP dataset (Figure S2, Supplemental Digital Content, https://links.lww.com/MD/Q305)

### 3.2. Sensitivity analysis

The results of sensitivity analysis confirmed the robustness of our main MR analysis. No heterogeneity was found among the 11 proteins. Measured by Cochran *Q* statistics (*P* > .05) according to the MR Egger intercept assessment, IVs did not have horizontal pleiotropy (Egger intercept *P* > .05) or MR-PRESSO comprehensive pleiotropy test (global test *P* > .05) (Table S2, Supplemental Digital Content, https://links.lww.com/MD/Q306). Two-way MR analysis did not find evidence of reverse cause (Table S3, Supplemental Digital Content, https://links.lww.com/MD/Q306) and Steige filtering ensured directionality (Table S4, Supplemental Digital Content, https://links.lww.com/MD/Q306). Our study observed strong evidence of co-localization between 5 proteins (REG1B, FLT4, IL6ST, GOLM1, and FAM3D) and DU (Table S5, Supplemental Digital Content, https://links.lww.com/MD/Q306; Figure S3, Supplemental Digital Content, https://links.lww.com/MD/Q305).

### 3.3. Mediation analysis

**Figure 5.**
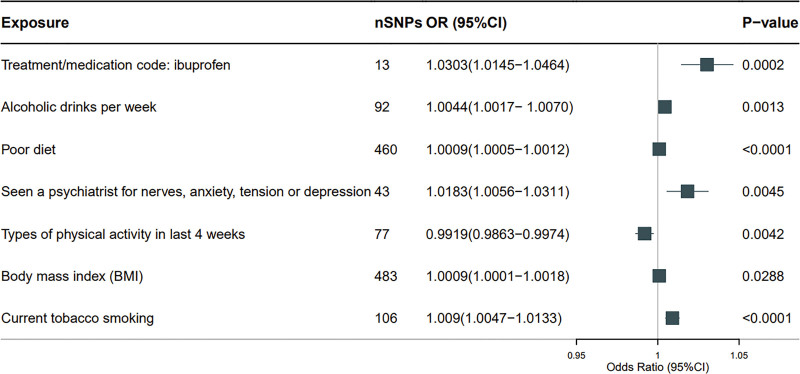
Forest plot of MR results for 7 risk factors and DU. A square is a causal estimate of OR values, and the horizontal line on the square represents the 95% confidence interval of these OR values. nSNPs: used to estimate the number of SNPs for causal effects in the graph. The *P*-value was determined by IVW nuclear magnetic resonance method. CI = confidence interval, DU = duodenal ulcer, IVW = variance weighted reciprocal, MR = Mendelian randomization, OR = odds ratio, SNPs = single nucleotide polymorphisms.

We excluded risk factors without GWAS data, such as genetic susceptibility and excessive gastric acid secretion. Subsequent MR analysis showed that there was no causal relationship between certain factors, leading us to focus further analysis on retaining factors. We founded treatment/medication code: ibuprofen (OR = 1.0303, 95% CI = 1.0145–1.0464, *P* = .0002), alcoholic drinks per week (OR = 1.0044, 95% CI = 1.0017–1.0070, *P* = .0013), poor diet (OR = 1.0009, 95% CI = 1.0005–1.0012, *P* = 3.19E−06), seen a psychiatrist for nerves, anxiety, tension, or depression (OR = 1.0183, 95% CI = 1.0056–1.0311, *P* = .0045), BMI (OR = 1.0009, 95% CI = 1.0001–1.0018, *P* = .0288), and current tobacco smoking (OR = 1.0090, 95% CI = 1.0047–1.0133, *P* = 3.84E−05) are increased risk of DU, while physical activity in the last 4 weeks (OR = 0.9919, 95% CI = 0.9863–0.9974, *P* = .0042) was associated with a reduced risk of DU (Fig 5; Table S6, Supplemental Digital Content, https://links.lww.com/MD/Q306). Subsequently, we investigated the causal relationship between the proteins identified by co-localization (REG1B, FLT4, IL6ST, GOLM1, and FAM3D) and risk factors (Table S7, Supplemental Digital Content, https://links.lww.com/MD/Q306). Our results showed that an increase in gene level of REG1B was associated with a decrease in alcoholic drinks per week (OR = 0.9752, 95% CI = 0.9577–0.9931, *P* = .0067) and poor diet (OR = 0.9832, 95% CI = 0.9708–0.9957, *P* = .0084). In addition, the higher the gene determined IL6ST level, the more alcoholic drinks per week (OR = 1.0126, 95% CI = 1.0024–1.0230, *P* = .0157). Conversely, the higher the gene determined GOLM1 level, the less alcoholic drinks per week (OR = 0.9822, 95% CI = 0.9684–0.9962, *P* = .0127). To explore the indirect impact of proteins on DU through risk factors, we conducted mediation analysis using 2-step MR effect estimation and the overall effect of major MR. The mediation effects of REG1B through daily alcoholic drinks per week and poor diet were 4.50% and 0.70%, respectively. The indirect impact of IL6ST on the risk of DU through alcoholic drinks per week accounts for 2.10%, while the indirect impact of GOLM1 on the risk of DU through alcoholic drinks per week accounts for 3.50% (Table S8, Supplemental Digital Content, https://links.lww.com/MD/Q306).

### 3.4. The association between potential drug targets and current DU drugs

**Figure 6.**
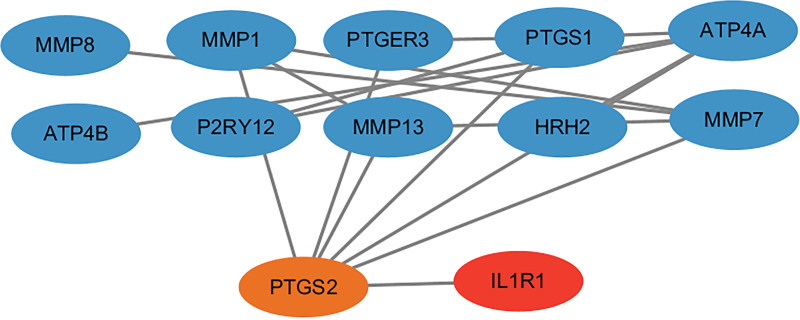
The protein-protein interaction network between DU-related proteins and current drug targets. The red circle represents plasma protein (IL1R1). The orange solid circle represents current DU drug therapy targets associated with potential proteins, while the blue solid circle represents current DU drug therapy targets without such association. DU = duodenal ulcer.

Based on the results from the Opentarget database, we identified 29 drugs associated with DU and 13 targets associated with current drugs (Table S9, Supplemental Digital Content, https://links.lww.com/MD/Q306). The PPI network revealed the interaction between the identified protein IL1R1 and the target of current DU drugs (Fig. 6). Specifically, IL1R1 is associated with PTGS2, which is a target of aspirin, celecoxib, diclofenac, diclofenac sodium, and naproxen.

### 3.5. Clinical stages of candidate protein targets and prediction of potential drugs

It is worth noting that the targeted drug alanyl glutamine targeting REG1B is entering inflammation, phase III trial of nutrition and enteropathy. FLT4-targeted drugs famitinib, catequentinib, sunitinib, and axitinib are undergoing phase I/II trials for various cancers, including nasopharyngeal carcinoma, renal cell carcinoma, colorectal adenocarcinoma, lung cancer, and prostate cancer. In addition, the IL6ST targeted drug siltuximab is undergoing I/II trials for solid tumors, while the EPHB4-targeted drug tesevatinib is undergoing I/II trials for solid tumors and non small cell lung cancer. The IL1R1-targeted drugs anakinra and lenvatinib are undergoing phase I/II/III trials for rheumatoid arthritis, pulmonary arterial hypertension, hydradenitis supplementation, and unstable stage III or stage IV melanoma (Table S10, Supplemental Digital Content, https://links.lww.com/MD/Q306). There are currently no ongoing experiments for IGSF3, GOLM1, DPEP2, FAM3D, QSOX2, and SEMA6A. However, DPEP2 and QSOX2 may still be potential targets. Next, we will use the DSigDB database to predict drugs that may target plasma protein targets for 7 druggable DU. The identified drugs include GW768505A GSK, Cabozantinib FDA, AZ-628 LINCS, PONATINIB CTD 00004976, WH-4-025 LINCS, Gefitinib, Ponatinib FDA, Sorafenib, PD-173955 Kinome Scan, Vandetanib, thalidomide CTD 00006858, HG-6-64-01 LINCS, EXEL-2880/GSK-1363089 Kinome Scan, ciglitazone CTD 00001835, 5-Fluorouracil BOSS, R406 Kinome Scan, TAE-684 Kinome Scan, OTSSP167 LINCS, AZD7762 LINCS, Sunitinib, tolnaftate PC3 UP, puromycin HL60 UP, and vatalanib succinate TTD 00011775. We speculate that these drugs may have potential therapeutic effects on DU by targeting identified plasma proteins.The information on candidate drug gene interactions obtained from the DSigDB database can be found in Table S11, Supplemental Digital Content, https://links.lww.com/MD/Q306.

## 4. Discussion

### 4.1. Key findings

We used plasma pQTL data to identify potential therapeutic targets for DU, revealing 11 proteins. The “causal relationship” determined by magnetic resonance imaging may be influenced by reverse causal relationships, horizontal pleiotropy, or confounding factors. Therefore, bidirectional MR was performed without protein showing reverse causal relationships, which is further supported by Steiger filtering. Co-localization analysis showed an association between gene predicted REG1B, FLT4, IL6ST, GOLM1, FAM3D, and DU levels (please refer Table S12, Supplemental Digital Content, https://links.lww.com/MD/Q306 for details). In addition, we have listed the developing drug-related protein targets for DU (IL1R1) and 5 pharmaceutically acceptable proteins (REG1B, IL6ST, FLT4, GOLM1, and EPHB4). Our research findings indicate that treatment/medication code: ibuprofen, alcoholic drinks per week, poor diet, seen a psychiatrist for nerves, anxiety, tension, or depression, types of physical activity in last 4 weeks: other exercises (e.g., swimming, cycling, keep fit, bowling), BMI, and current tobacco smoking are causally related to DU, highlighting their role in the pathogenesis of DU. Studies have shown that long-term or massive use of ibuprofen will significantly increase the incidence rate of DU.^[40]^ Smoking and alcohol consumption increase the risk of developing DU.^[41]^ Some studies have suggested that diet, emotional stress, and anxiety can affect the occurrence and development of DU.^[42,43]^ Cheng et al found that physical activity can provide a nondrug method to reduce the incidence rate of male DU.^[44]^

### 4.2. Potential protein targets of DU

The human regenerative gene 1B (REG1B) belongs to the type I subclass of the REG family and was first discovered in 1993. It consists of 6 exons and 5 introns, encoding a protein of 166 amino acids, and is typically expressed in the gastrointestinal mucosa. Recently, it has been found that REG1B plays an important role in intestinal health and may be a putative indicator of intestinal injury and repair. The formation of DU is closely related to the destruction of mucosal barriers, and REG1B may participate in the healing process of ulcers by promoting the regeneration and repair of mucosal cells.^[45,46]^ According to reports, the expression of REG1B may be influenced by the gut microbiota, especially in cases of inflammation or infection (such as *H pylori* infection).^[47]^ Therefore, REG1B may affect the progression of ulcers by regulating immune responses and mucosal repair. In our study, REG1B was identified as a protective factor for DU, which was validated in additional GWAS data and co-localization analysis. In addition, our study further found that individuals with a history of alcohol consumption and poor diet have lower levels of REG1B in their bodies. People who drink alcohol and have poor diet may express lower levels of REG1B, leading to weakened repair ability of intestinal mucosa after injury, reducing the role of maintaining gastrointestinal mucosal integrity, and thus affecting DU. Although our drug target prediction did not predict drugs targeting REG1B, it is worth noting that REG1B is currently undergoing clinical trials for inflammation, nutrition, and enteropathy. This indicates that REG1B is more likely to be associated with DU. Future research can focus on elucidating the mechanism by which REG1B is involved in the development of DU, particularly in the populations of drinkers and those with poor diets. These studies may help in targeted treatment of DU.

IL-1 receptor type 1 (IL-1R1) is a receptor of the interleukin-1 (IL-1) pathway, through which IL-1 signals and appears as a central mediator of systemic inflammation.^[48]^ IL1R1 signaling induces a reactive state called intestinal gliosis, characterized by strong induction of different chemokines, cytokines, and colony-stimulating factors 1 and 3, leading to intestinal inflammatory response.^[49]^ Long-term intestinal inflammation, especially duodenitis, damages the intestinal mucosa, weakens defense mechanisms, and leads to ulcers.^[50]^ Our study suggests that IL1R1 is a risk factor for DU and provides evidence of its interaction with the current drug target PTGS2 for DU, suggesting that IL-1R1 is more likely to be a target protein for DU. In addition, it is worth noting that IL-1R1 is currently undergoing clinical trials for diseases such as rheumatoid arthritis, hydradenitis supurativa, pulmonary arterial hypertension, melanoma, etc and it is predicted that IL-1R1 can serve as a target for drugs such as thalidomide CTD 00006858, ciglitazone CTD 00001835, 5-fluorouracil BOSS, puromycin HL60 UP, and mebendazole HL60 UP. In the future, the therapeutic potential of IL-1R1 in DU can be further explored, and the development of drugs targeting it may bring new therapeutic breakthroughs.

The IL6ST/gp130 signal transducer of the interleukin-6 cytokine family, recruited from the LIF/IL-6 cytokine family, can induce tissue damage by initiating neutrophil aggregation, inducing transcriptional inflammatory responses, and producing harmful products, reactive oxygen species, and lysosomal enzymes that cause gastrointestinal tissue damage.^[51]^ Intestinal ulcers may be caused by a reduction in the first line of defense against mucosal surface pathogens mediated by STAT dependent IL-6 and impaired epithelial homeostasis.^[52,53]^ In our study, IL6ST was identified as a risk factor for DU and co-localization analysis was performed. In addition, our research further found that individuals who consume alcohol have higher levels of IL6ST in their bodies. IL6ST plays a central role in IL-6 signaling, and alcohol intake may upregulate IL6 expression, enhance IL-6ST signaling, and further promote inflammation.^[54,55]^ However, establishing a direct link between IL6ST and DU requires further investigation. IL6ST may affect DU through inflammatory pathways, involvement in affecting mucosal barrier function and repair ability, and its association with alcohol induced changes. In the future, the mechanism of IL6ST occurrence in DU and its interaction with alcohol induced factors can be explored to investigate its impact on DU. In addition, it is worth noting that IL6ST is currently undergoing clinical trials for solid tumors. And it is predicted that IL6ST can serve as a target for drugs such as thalidomide CTD 00006858, ciglitazone CTD 00001835, and puromycin HL60 UP.

Tyrosine kinase EphB4 is the largest receptor in the EphB subfamily and, like all other Eph receptors, is a type I transmembrane protein with a prototype RTK topology. Therefore, the protein is characterized by an N-terminal multi-domain extracellular region, a single transmembrane fragment, and a cytoplasmic region containing a C-terminal kinase domain. The extracellular domain includes a globular ligand binding domain, a cysteine rich region, and is composed of an epidermal growth factor-like motif and 2 fibronectin type III repeat sequences.^[56,57]^ EphB4 protein is widely expressed in proliferating stratified intestinal epithelial cells^[58]^ and can participate in the regulation of immune responses, as well as the remodeling and maintenance of intestinal epithelial layer integrity.^[58]^ According to reports, EphB4 can promote the occurrence and development of intestinal inflammation by regulating the inflammatory signaling pathway and the release of pro-inflammatory factors.^[59]^ There is a significant positive correlation between it and macrophage migration inhibitory factor, which can promote gastrointestinal inflammation associated with *H pylori* infection.^[60]^ Although the direct relationship between EPHB4 and DU is not fully understood, its role in inflammation regulation, cell proliferation, and apoptosis suggests that it may play an important role in ulcer formation and occurrence, but the specific mechanism still needs further research. Our research findings suggest that EPHB4 may actively promote the development of DU and have been validated in other GWAS data. Further research results indicate that EPHB4 is currently undergoing clinical trials in solid tumors and non-small cell lung cancer. EPHB4 is expected to serve as targets of drugs such as GW768505A GSK, Cabozantinib FDA, AZ-628 LINCS, PONATINIB CTD 00004976, WH-4-025 LINCS, gefitinib, linatinib FDA, sorafenib, PD-173955 Kinome Scan, and Vandetanib etc.

DPEP2 belongs to the membrane-bound dipeptidase (Dpep) family and is a cell membrane enzyme anchored to glycosylphosphatidylinositol. It is responsible for hydrolyzing dipeptides, including leukotriene D4, the beta lactam ring of certain antibiotics, and cysteine bis-gly formed during glutathione degradation. Dpep expression changes in the context of inflammatory diseases, indicating that they may be related to immune responses.^[61,62]^ Currently, there is no research directly linking DPEP2 to DU, but some studies have found that DPEP2 is a central gene in colorectal adenocarcinoma that can affect the prognosis of colorectal cancer.^[63]^ Our study found that DPEP2 is a risk factor for DU and has been validated in other GWAS data. Furthermore, we predicted that DPEP2 could serve as a target for tolnaftant PC3 UP drugs. This indicates that DPEP2 has the potential to serve as a pathogenic protein for DU.

QSOX2 (Quiescin thiol oxidase 2, MIM 612860), a multi-domain enzyme belonging to the thiol oxidase family, most notably catalyze the introduction of disulfide bonds in secreted proteins.^[64]^ QSOX2 deficiency can lead to gastrointestinal motility disorders, but the unique role of QSOX2 in gastrointestinal physiology remains to be elucidated.^[65]^ In our study, we found that QSOX2 is associated with reducing DU, but no drugs related to it were predicted. In the future, we can further investigate the specific molecular pathways and genetic background of QSOX2 in digestive diseases.

### 4.3. Emerging proteins with therapeutic potential

Currently, 4 proteins (IGSF3, FAM3D, SEMA6A, GOLM1) have not been listed as drug targets. IGSF3 function contributes to the formation of the enteric nervous system. Given the important role of the enteric nervous system in maintaining normal gastrointestinal function, our study provides additional information needed to further understand the mechanisms of enteric nerve innervation and the etiology behind intestinal motility disorders.^[66]^ The intestinal secreted protein FAM3D plays an important role in maintaining intestinal homeostasis, preventing inflammation-related cancers, and maintaining normal microbial composition.^[67]^ SEMA6A is a multifunctional transmembrane signaling protein involved in various cellular processes, including axonal guidance, cell migration, and cancer progression.^[68]^ Golgi apparatus membrane protein 1 (GOLM1) is a type 2 transmembrane protein of the Golgi apparatus, which is associated with inflammation and immune regulation. The reduction of goblet cells lacking mucus in GOLM1 disrupts intestinal homeostasis, leading to the breakdown of the intestinal barrier and exacerbating intestinal diseases.^[69]^ Our study found that the level of GOLM1 is negatively correlated with DU and co-localized. The intermediary analysis results show that individuals with a history of alcohol consumption have lower levels of GOLM1 in their bodies. It is suggested that drinkers may express lower levels of GOLM1, leading to disruption of intestinal homeostasis and thus affecting the occurrence and development of DU. There is no evidence of interaction between GOLM1 and current drug targets for DU, but GOLM1 may be a pathogenic protein for DU. There is currently no direct research on the association between IGSF3, FAM3D, GOLM1, and SEMA6A and DU. Future research should focus on elucidating the specific molecular mechanisms of these proteins in DU, exploring their potential as therapeutic targets, and developing relevant therapeutic drugs.

### 4.4. Strengths

Our study utilized dual sample MR and Bayesian co-localization analysis, as well as drug target assessment. We identified 11 plasma proteins that are causally associated with DU, of which 5 proteins were co-localized and 7 proteins were considered as drug genes. This emphasizes the effectiveness of our method in elucidating the fundamental mechanisms underlying the pathogenesis of DU. Our research is based on genetic screening of biomarkers associated with DU, laying the foundation for personalized prevention strategies for individuals at genetic risk, thereby improving the targeting and effectiveness of treatment.

### 4.5. Limitations

But we must acknowledge some limitations of our research. Firstly, the GWAS summary data used for MR comes from a European population lacking summary data from other regions, which has population specificity with regional and racial limitations. Future research, especially on the universality beyond European ancestors, is crucial for verifying these associations. Secondly, caution should be exercised when interpreting the PPH4 in collaborative localization, as a lower PPH4 may not necessarily indicate a lack of evidence supporting collaborative localization, especially when PPH3 is also lower due to its limited statistical power. Perhaps this problem can be addressed by enhancing the capabilities of existing co-localization methods through improved analysis estimation, fine drawing methods, and explicit modeling of different LD patterns across datasets.^[70]^ Thirdly, we used plasma protein deCODE data from the Icelandic population in Europe as the exposure level, and DU data obtained from the UK Biobank as the result for primary MR analysis. Our results were validated in UKBPPP and other GWAS data, but could not be validated as QSOX2, GOLM1, and SEMA6A proteins were not detected in the UKBPPP dataset. Fourthly, this study identified the drug-related protein target (IL1R1) and 6 pharmaceutically acceptable proteins (REG1B, IL6ST, FLT4, DPEP2, QXOS2, and EPHB4) for DU. However, we must recognize that this only provides suggestive results, not clear conclusions. Therefore, further research is needed to thoroughly verify the above relationship. Finally, our study revealed that the limited genetic prediction of REG1B, IL6ST, and GOLM1, mediated by alcohol intake and poor diet, has an impact on DU. Additional research is also needed to explore other potential mediators.

## 5. Conclusion

In summary, our genetic association study indicates a causal relationship between the 11 plasma proteins (REG1B, FLT4, IGSF3, IL6ST, GOLM1, EPHB4, DPEP2, FAM3D, QSOX2, SEMA6A, and IL1R1) determined by genes and DU. Especially IL1R1 and 6 pharmaceutically acceptable proteins (REG1B, IL6ST, FLT4, DPEP2, QXOS2, and EPHB4) show promising potential as therapeutic targets for DU. Further research is needed in the future to elucidate the mechanisms of action of these candidate proteins in DU.

## Acknowledgments

We thank the deCODE (https://www.decode.com/summarydata/), IEU Open GWAS Project (https://gwas.mrcieu.ac.uk/), and UK Biobank (https://www.ukbiobank.ac.uk/) for providing GWAS summary statistics data for our analysis.

## Author contributions

**Conceptualization:** Dan Luo.

**Data curation:** Chenhao Liu.

**Formal analysis:** Huize Zhang.

**Methodology:** Simin Cao.

**Visualization:** Mingyue Long.

**Writing – original draft:** Xu Luo.

**Writing – review & editing:** Yi Liu.

## Supplementary Material




