# Supplementary material for: Employing plasma proteins in proteomic Mendelian randomization analysis to identify therapeutic targets for duodenal ulcer
Source: Medicine (Baltimore). 2025 Oct 31;104(44):e45093. doi: 10.1097/MD.0000000000045093 (PMC12582692; doi:10.1097/MD.0000000000045093)
Supplement: Supplementary file 2 [file medi-104-e45093-s002.docx]

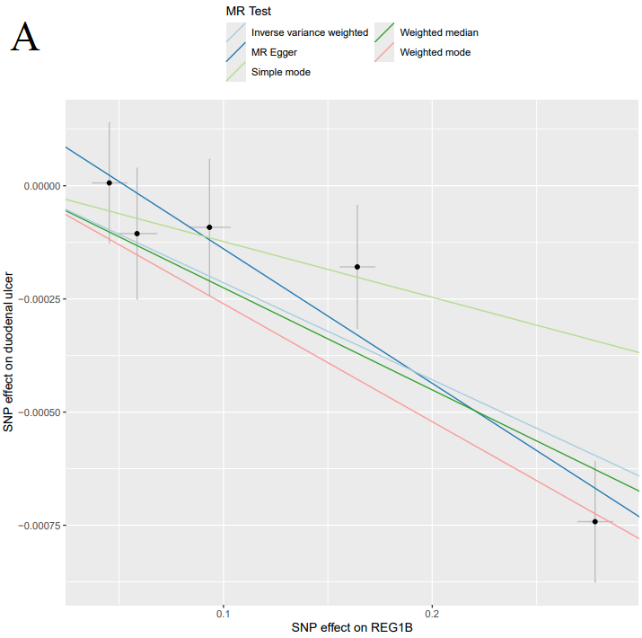

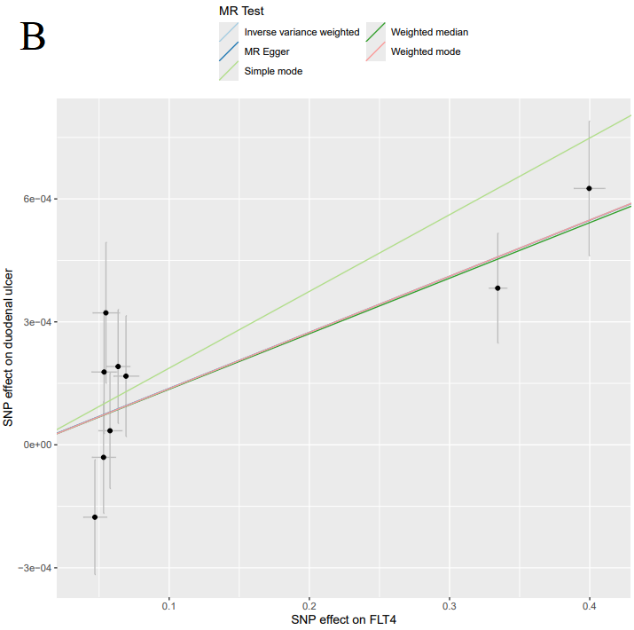

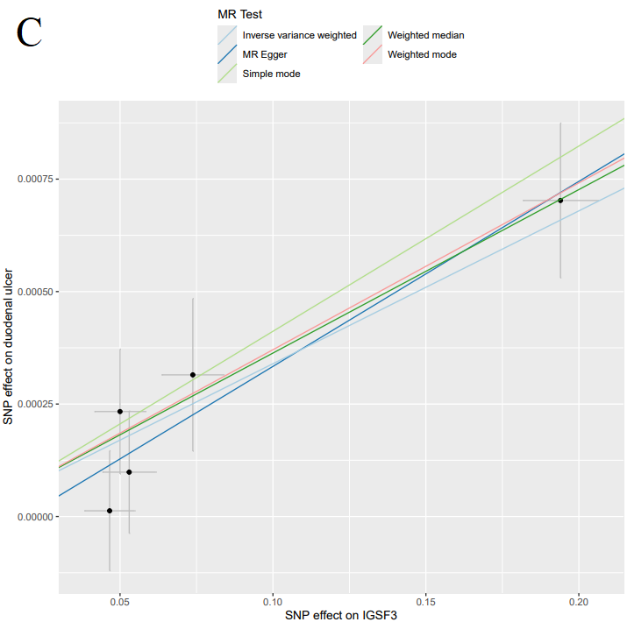

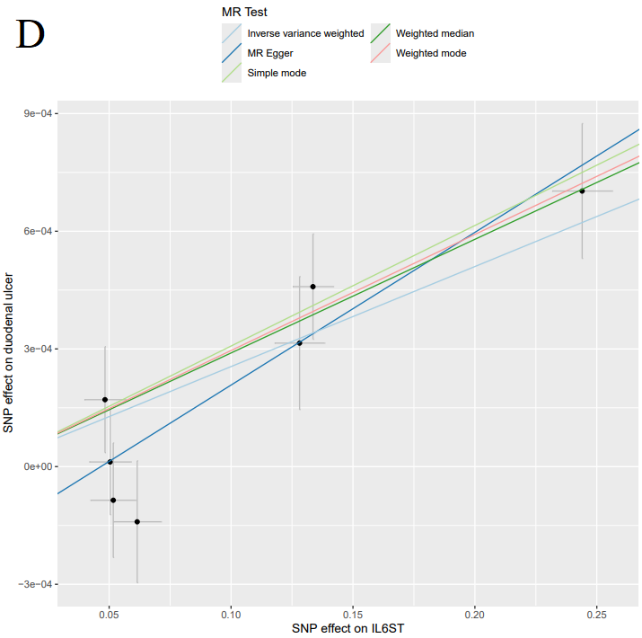

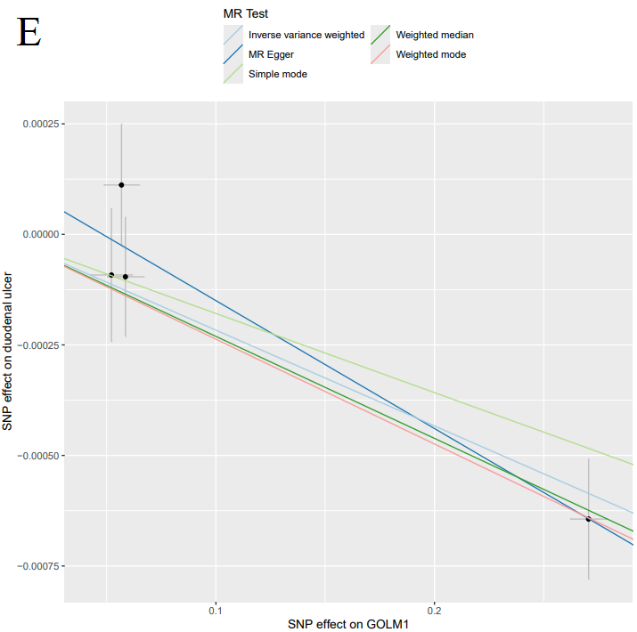

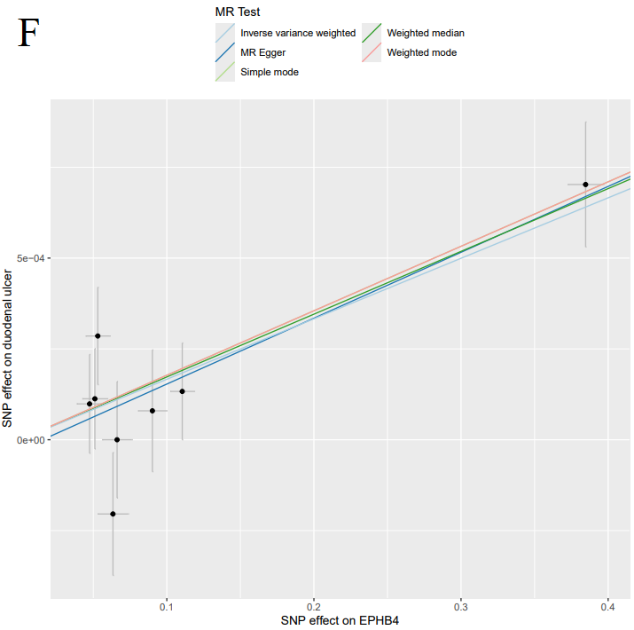

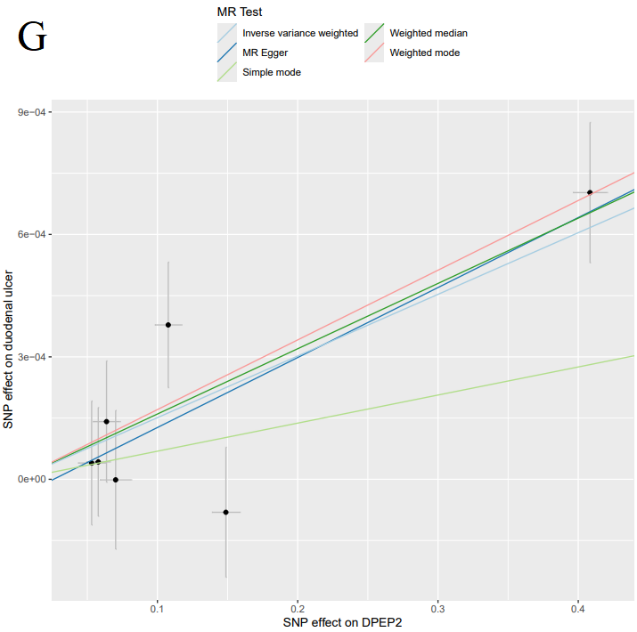

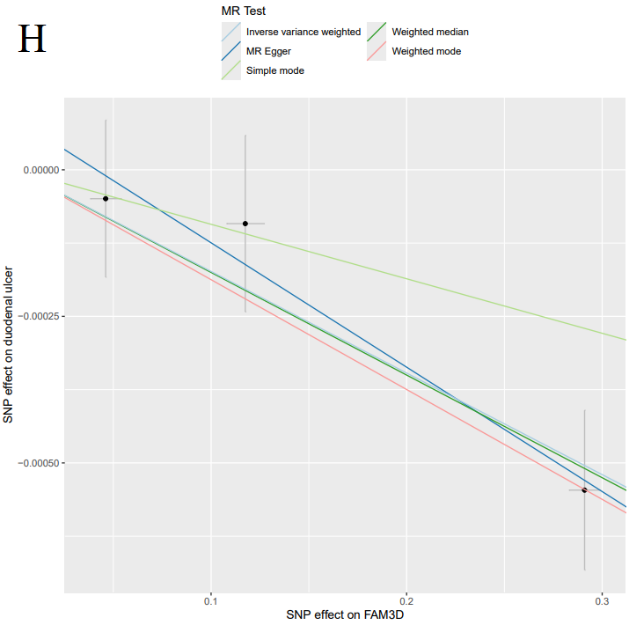

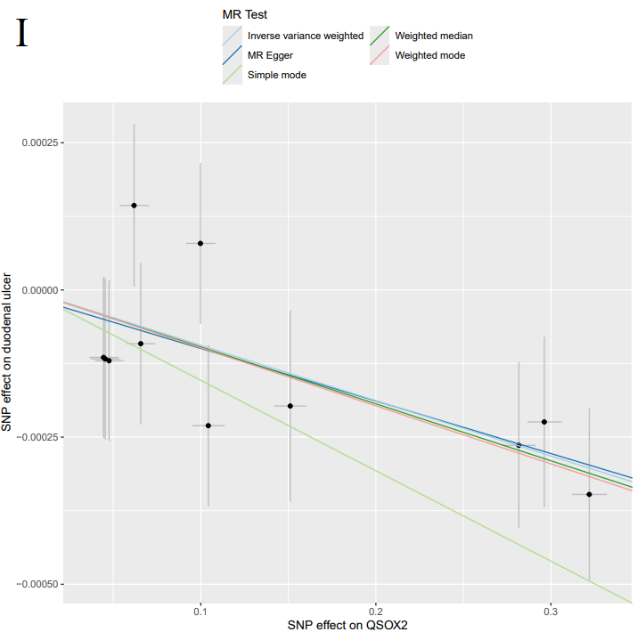

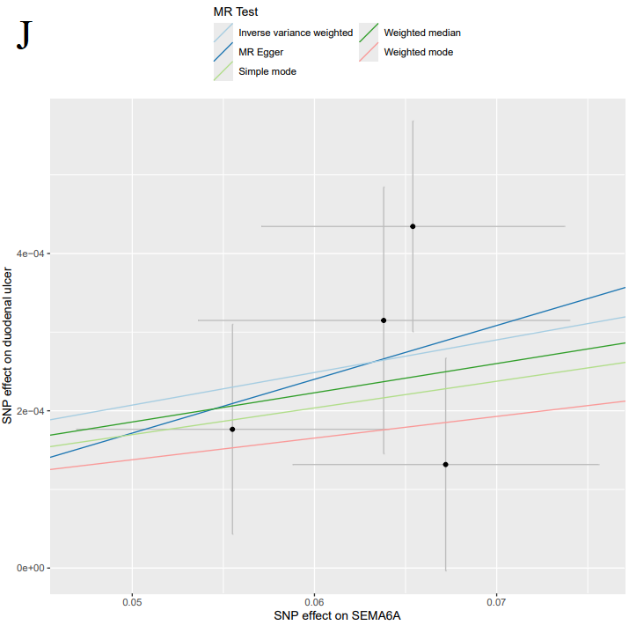

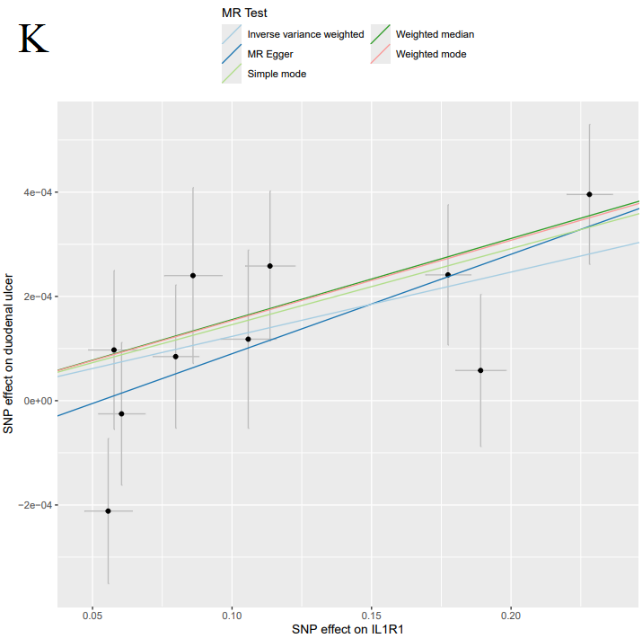


**Fig S1** Standard mendelian randomization plots of plasma proteins and Duodenal ulcer. The different regression lines indicated the effect sizes as calculated by different mendelian randomization tests (methods). A: REG1B: regenerating family member 1 beta ;B: FLT4: fms related receptor tyrosine kinase 4;C:IGSF3: immunoglobulin superfamily member 3;D:IL6ST: interleukin 6 cytokine family signal transducer E:GOLM1: golgi membrane protein 1;F:EPHB4: EPH receptor B4; G:DPEP2: dipeptidase 2;H:FAM3D: FAM3 metabolism regulating signaling molecule D; I:QSOX2: quiescin sulfhydryl oxidase 2; J:SEMA6A : semaphorin 6A ;K:IL1R1 : interleukin 1 receptor type 1.


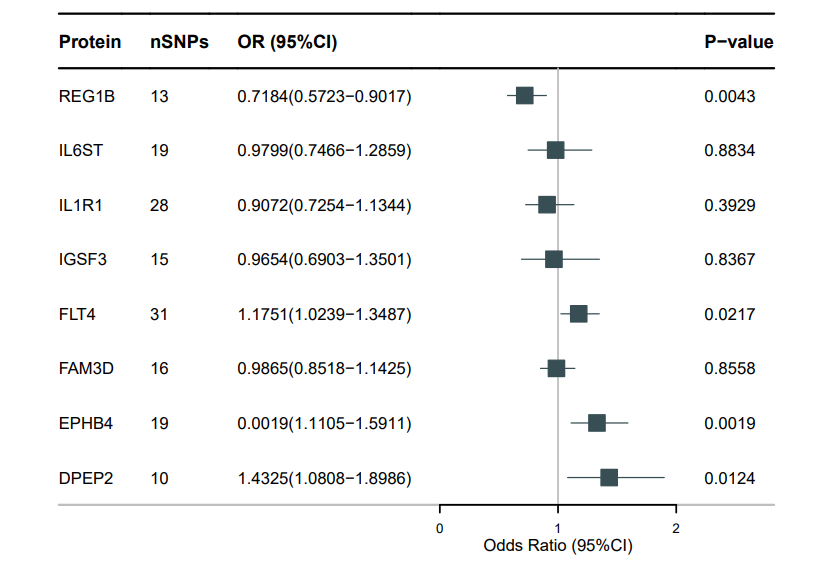


**Fig S2 externally validates the causal relationship between eight potential pathogenic proteins and duodenal ulcer through MR analysis.** A square is a causal estimate of OR values, and the horizontal line on the square represents the 95% confidence interval of these OR values. nSNPs: Used to estimate the number of SNPs for causal effects in the graph. The P-value value was determined by IVW nuclear magnetic resonance method. OR: Odds ratio; 95% CI: 95% confidence interval; IVW: Variance weighted reciprocal.


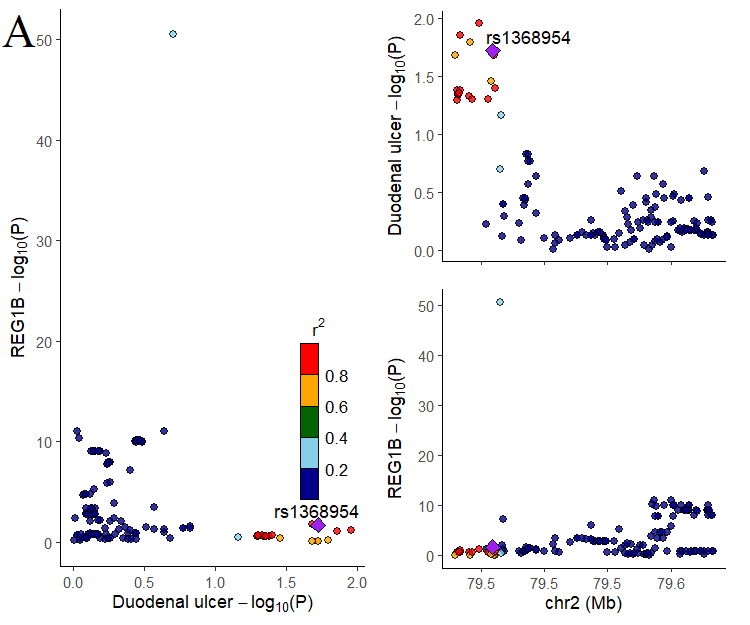

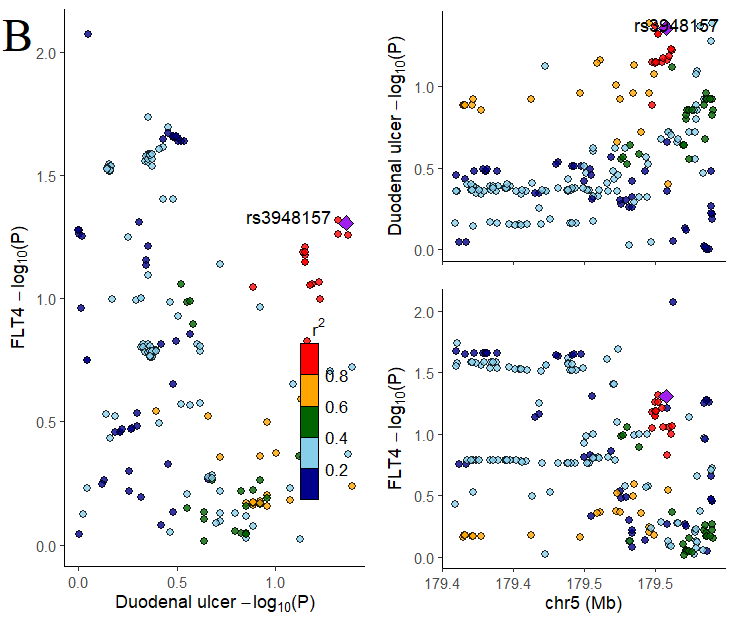

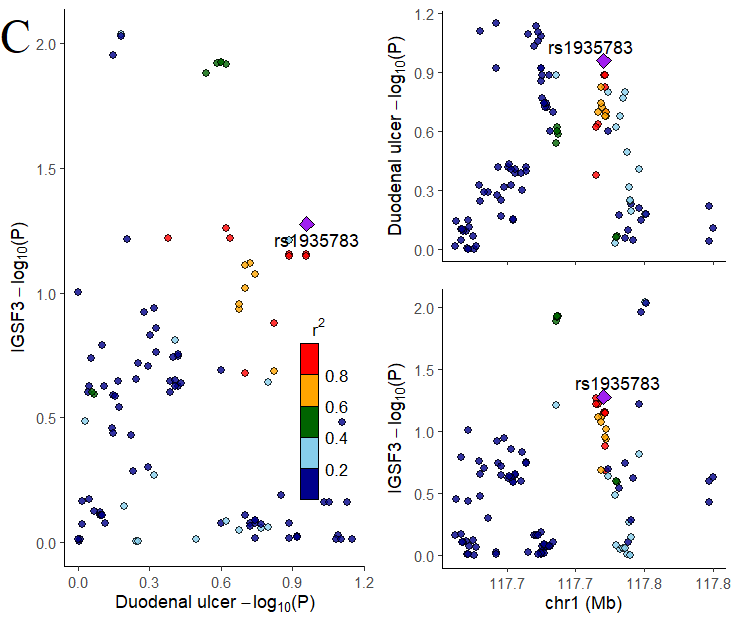

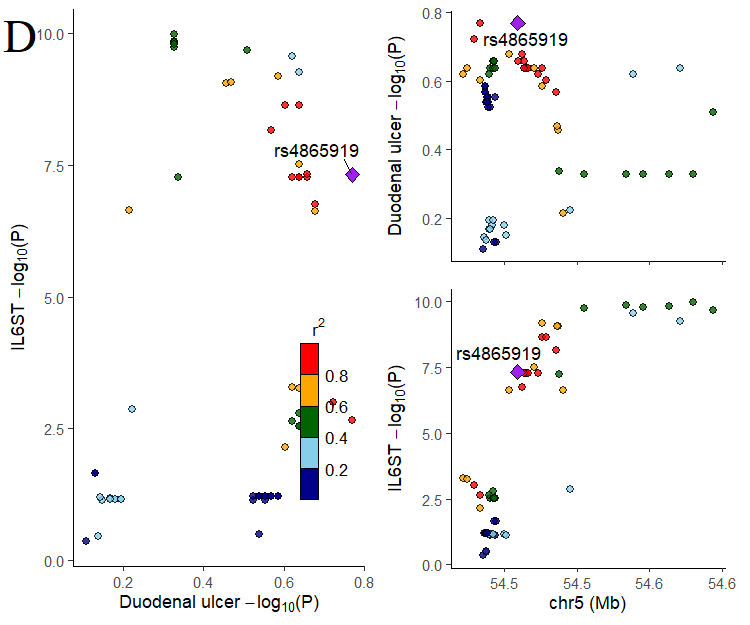

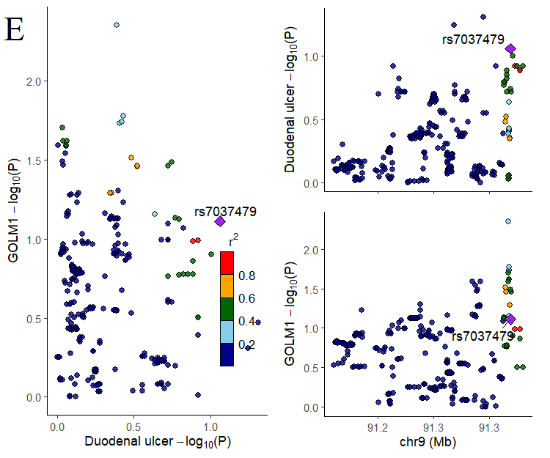

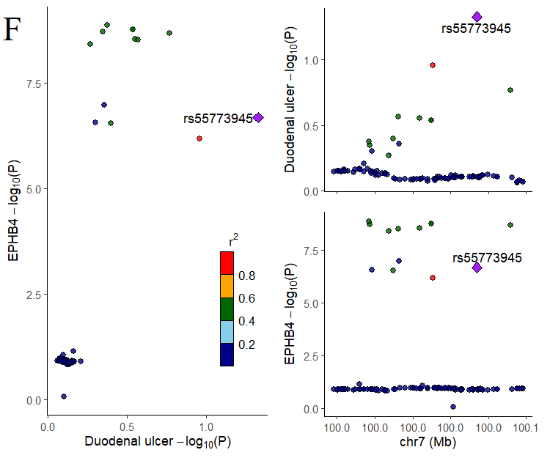

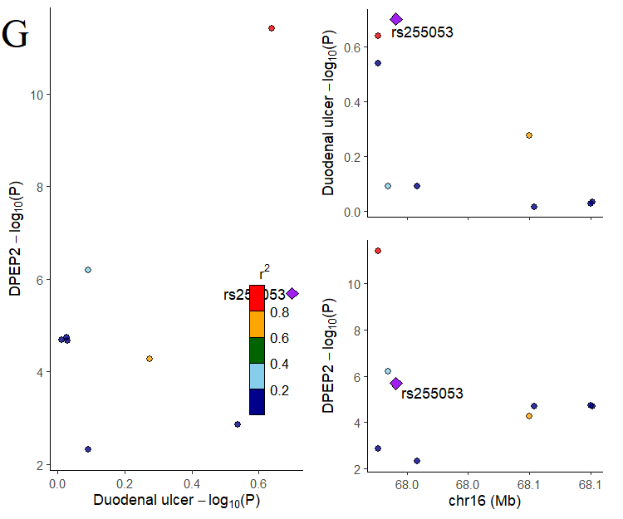

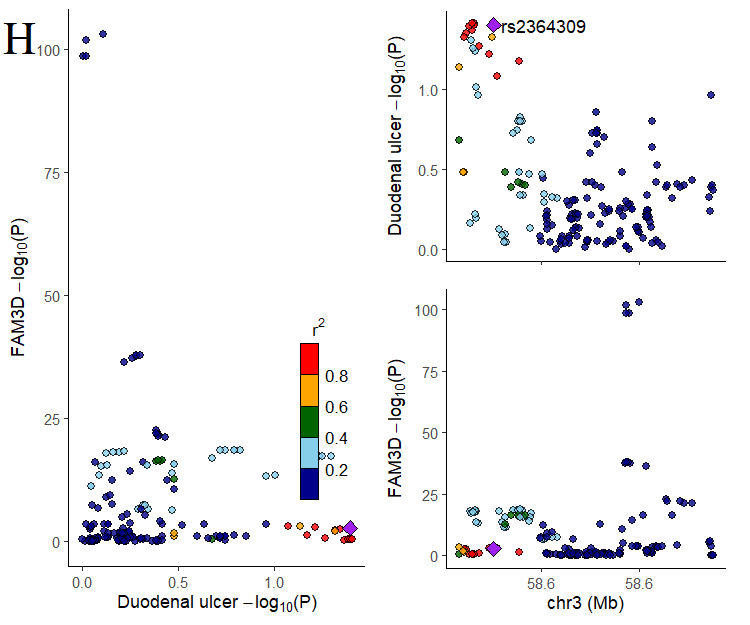


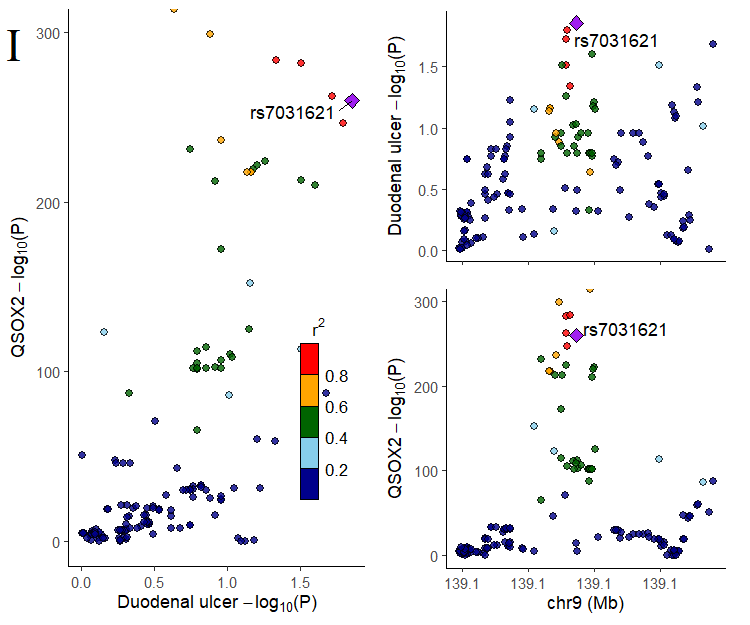

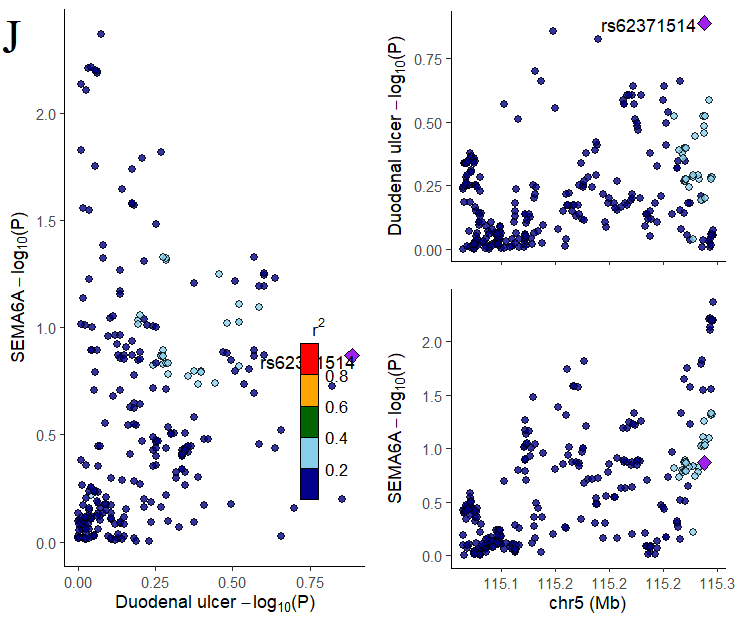

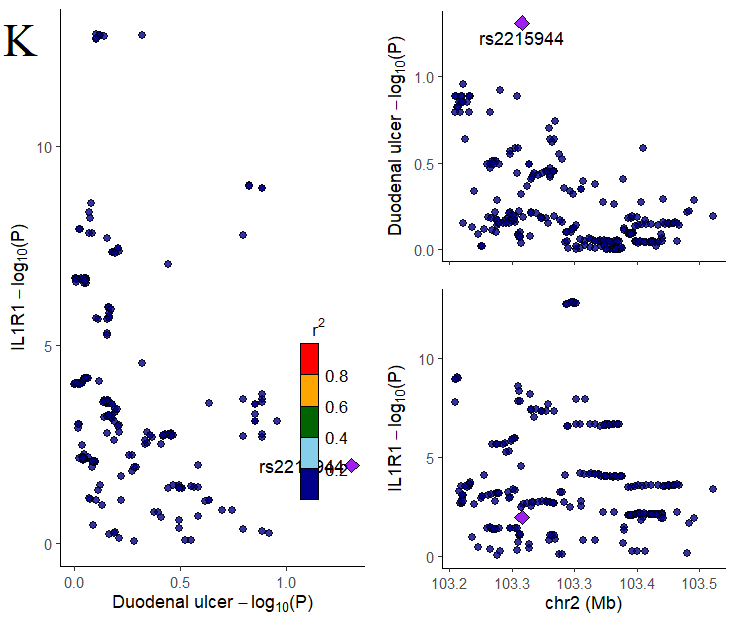


**Fig S3 Co-localization plots of pQTLs and genetic associations of duodenal ulcer.** A: REG1B: regenerating family member 1 beta ;B: FLT4: fms related receptor tyrosine kinase 4;C:IGSF3: immunoglobulin superfamily member 3;D:IL6ST: interleukin 6 cytokine family signal transducer E:GOLM1: golgi membrane protein 1;F:EPHB4: EPH receptor B4; G:DPEP2: dipeptidase 2;H:FAM3D: FAM3 metabolism regulating signaling molecule D; I:QSOX2: quiescin sulfhydryl oxidase 2; J:SEMA6A : semaphorin 6A ;K:IL1R1 : interleukin 1 receptor type 1.
